# Comparison of different deconvolution algorithms for voxel-wise quantitative MR perfusion assessment

**DOI:** 10.1186/1532-429X-13-S1-P59

**Published:** 2011-02-02

**Authors:** Niloufar Zarinabad Nooralipour, Amedeo Chiribiri, Gilion Hautvast, Andreas Schuster, Philip Batchelor, Sven Plein, Eike Nagel

**Affiliations:** 1 Division of imaging Sciences, King's College London, London, UK; 2 Wellcome Trust – EPSRC Centre of Excellence in Medical Engineering, London, UK; 3 Philips Healthcare, Imaging Systems - MR, Best, Netherlands; 4 D7University of Leeds, Multidisciplinary Cardiovascular Research Centre and Division of Imaging Sciences, King’s College London, Leeds-London, UK; 5 Di1King’s College London, Wellcome Trust – EPSRC Centre of Excellence in Medical Engineering, London, UK

## Objective

To apply deconvolution algorithms to voxel-wise analysis of first-pass myocardial perfusion MR data and to determine how noise affects perfusion estimation using different quantification methods.

## Background

One of the main advantages of cardiac first-pass perfusion MR is its high spatial resolution. Though several methods have been used to quantify myocardial perfusion rate, no previous work has been done on voxel-wise analysis and published methods were applied to standard myocardial segments. Signal-to-noise ratio (SNR) might negatively affect the accuracy of the measurements obtained with methods developed for segmental analysis.

## Methods

**Figure 1:**
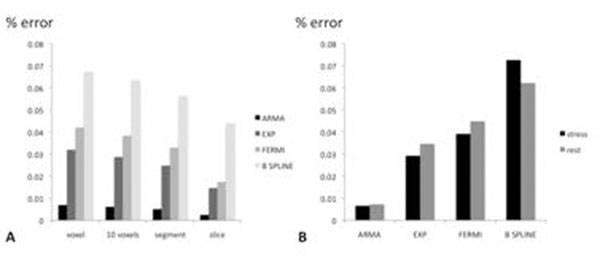
Comparison of relative curve fit error for different levels of SNR for ARMA, exponential, Fermi and B-spine deconvolution (A). Relative error was found to be higher for rest perfusion images (B).

Fermi function modelling, auto-regressive moving-average model (ARMA), model-independent approach using B-spline-basis and exponential-basis deconvolution were used to quantify myocardial perfusion rate in 6 patients. Perfusion data were acquired on a 3 Tesla Philips CMR scanner in three LV short axis slices with a saturation recovery gradient echo method (TR/TE 3.0ms/1.0ms, flip-angle 15°; effective kt-SENSE acceleration 3.8, spatial resolution 1.2x1.2x10mm) during adenosine-induced hyperaemia (140µg/kg/min) using 0.05mmol/kg Gd-DTPA (Magnevist, Schering, Germany) at 4ml/minute followed by a 20 ml saline flush. Voxel-wise perfusion estimates were compared to the results obtained from segmental analysis, for different levels of spatial averaging (10 voxels - 1 segment - whole slice). The data shown in Figure 1 represent the curve fit relative error, the square root of the sum of squared difference between the measured tissue data and the estimated tissue data, computed by convolving the measured blood data with the estimated tissue kernel, divided by the norm of measured tissue data.

## Results

ARMA method analysis resulted in the lowest model curve-fit error for different level of noise in both rest and stress condition. Exponential deconvolution had a lower error when compared to Fermi function modelling and model independent analysis (Figure 1A). The overall rest and stress error was in the voxel-wise/segment-wise analyses 0.6%/0.5% for ARMA, 3.2/2.4% for exponential, 4%/3.3% for Fermi, and 7%/5.6% for B-spline deconvolution, respectively.

The curve-fit error increased when analyzing rest perfusion images, most likely as a result of the increased baseline signal intensity values or of a lower perfusion rate in the rest images, acquired after stress.

## Conclusion

This study confirms the importance of adequate SNR in first-pass perfusion images. Before voxel-wise analysis can be used in clinical practice, more studies will be needed to define the best algorithms to deal with reduced SNR typical for voxel-wise tissue data. ARMA approach and Exponential basis deconvolution were the least sensitive methods to noise.

